# Estimates of Social Contact in a Middle School Based on Self-Report and Wireless Sensor Data

**DOI:** 10.1371/journal.pone.0153690

**Published:** 2016-04-21

**Authors:** Molly Leecaster, Damon J. A. Toth, Warren B. P. Pettey, Jeanette J. Rainey, Hongjiang Gao, Amra Uzicanin, Matthew Samore

**Affiliations:** 1 Department of Internal Medicine, University of Utah, Salt Lake City, Utah, United States of America; 2 Veterans Affairs Salt Lake City Health Care System, Salt Lake City, Utah, United States of America; 3 Department of Mathematics, University of Utah, Salt Lake City, Utah, United States of America; 4 Department of Global Migration and Quarantine, Centers for Disease Control and Prevention, Atlanta, Georgia, United States of America; 5 Department of Biomedical Informatics, University of Utah, Salt Lake City, United States of America; Melbourne School of Population Health, AUSTRALIA

## Abstract

Estimates of contact among children, used for infectious disease transmission models and understanding social patterns, historically rely on self-report logs. Recently, wireless sensor technology has enabled objective measurement of proximal contact and comparison of data from the two methods. These are mostly small-scale studies, and knowledge gaps remain in understanding contact and mixing patterns and also in the advantages and disadvantages of data collection methods. We collected contact data from a middle school, with 7th and 8th grades, for one day using self-report contact logs and wireless sensors. The data were linked for students with unique initials, gender, and grade within the school. This paper presents the results of a comparison of two approaches to characterize school contact networks, wireless proximity sensors and self-report logs. Accounting for incomplete capture and lack of participation, we estimate that “sensor-detectable”, proximal contacts longer than 20 seconds during lunch and class-time occurred at 2 fold higher frequency than “self-reportable” talk/touch contacts. Overall, 55% of estimated talk-touch contacts were also sensor-detectable whereas only 15% of estimated sensor-detectable contacts were also talk-touch. Contacts detected by sensors and also in self-report logs had longer mean duration than contacts detected only by sensors (6.3 vs 2.4 minutes). During both lunch and class-time, sensor-detectable contacts demonstrated substantially less gender and grade assortativity than talk-touch contacts. Hallway contacts, which were ascertainable only by proximity sensors, were characterized by extremely high degree and short duration. We conclude that the use of wireless sensors and self-report logs provide complementary insight on in-school mixing patterns and contact frequency.

## Introduction

In-person contacts create networks representing frequency of social mixing and routes of possible infectious disease transmission. Analysis of these networks can be used to better understand social interaction patterns and related behaviors [1] and the spread of disease [2–9]. Disease spread can be associated with the number of different contacts, the frequency and duration of contacts, as well as the qualitative nature of these contacts. These network characteristics frequently vary by both age and gender. Analysis of networks in the school-aged population, therefore, can be very informative for understanding and preventing infectious disease transmission, especially influenza and other acute respiratory infections. Children account for a large number of outpatient influenza or influenza-like illness visits, with increased morbidity and mortality associated with infection[10, 11], and are often the source of secondary family infections [12]. Influenza, and a number of other acute respiratory infections, is spread primarily through large droplets expelled through coughing, sneezing, or talking from the mouth of an infectious person directly into the eyes, nose, or mouth of a susceptible person and also via; 1) smaller, aerosolized particles inhaled by a susceptible person; and/or 2) hand-to-face self-inoculation by a susceptible person who has touched a contaminated, infectious person directly or a contaminated surface or object [13, 14]. While the relative importance of transmission modes remains unclear, frequency and duration of contacts within close proximity < 6 feet are thought to be of high relevance for influenza transmission. In practice contacts are defined to meet this or other definitions but there is no assignment of causation to modeled associations between specific contact definitions and transmission [15]. Common definitions of contact specify properties of intensity and duration. The intensity of a contact generally refers to proximity, physical contact, or talking. Different measurement approaches, by their nature, record contacts within specific definitions of intensity and duration. Self-reported contact definitions generally focus on talking and/or touching, spending time within arm’s reach of someone, and/or conversing for a specified duration [4, 16, 17]. On the other hand, wireless sensors will record data on proximal contact between people within a defined distance (e.g., 6 feet) for a set duration (as little as a few seconds), without respect to talking or touching. Logically, many proximal contacts (as recorded by sensors) also involve talking and/or touching (as reported in logs) and vice versa. However, some contacts only meet criteria for one definition and not the other.

There is a body of previous work that describes school-aged networks. Many of these are based on diaries or surveys. The POLYMOD study [16] was the first large-scale contact survey of its kind to provide estimates of contact frequency between age groups. There were over 7000 participants and the survey included information about location and timing of contact (e.g. weekdays, weekends, and holidays). Another large-scale study [3] collected contact information within 11 primary schools in Britain. Students were asked to name those they spent most time with in their class and in other classes. These and others [4, 18] are focused on self-report data from school-age children. There are at least two examples that use approximations of school-based social interactions based on schedules and surveys about contact and friendship. Using questionnaires, seating charts, and school schedules, [19] investigated and summarized mixing patterns in an elementary school. Statistical models were developed in [20] to estimate a high school contact network based on friendship network data as well as a contact survey that accounted for classroom structure. More recently, the use of remote sensors and other technologies has provided new and additional approaches for obtaining objective data on contact rates and social mixing. A full deployment of sensors in a high school [21] provided contact estimates and highlighted the importance of duration of contacts in understanding the network, subgroups, and assortativity, especially in support of transmission models. In another study, sensors were used over several days in two different years to collect contact data from a subset of high school classes [22] in order to provide estimated contact rates over time. A primary school sensor deployment [23] over two days collected data on 242 individuals in support of understanding transmission dynamics.

Measurement approaches have benefits as well as limitations. Self-report data have the benefit of being relatively easy to collect, with broad time and space dimensions; people are asked to recall what happened earlier at different venues or settings [2, 4, 16, 17, 24–26]. Limitations are related to recall bias and non-response. One study estimated the reporting accuracy of contact diaries [27] among 50 adults over three weeks by securing 100% participation and reporting of full names. Use of radio-frequency identification and received signal strength indication (RSSI) wireless sensing have the advantage of recording objective measurement of proximity[22, 28–30]. These technologies have the limitation to only people wearing them and in their performance in context of human behavior (people may not wear them as directed and certain behaviors shield sensor signals) [26, 31].

Studies to compare and contrast these approaches to measuring contact provide additional insight on both the benefits and limitations and also guide use of data in transmission models. Sensor data were compared to observed data on frequency and duration of contact between healthcare workers and patients [32]. Although they did not assess probability of capturing a contact via sensors, they reported that uncertainty in observed contact duration increased with duration of contact. In a study very similar to ours but in a high school, [33] data were collected using wireless sensors and contact surveys. They use these data to estimate nodal degree and reporting probabilities from the methods and put estimates from the two approaches into the context of the how contact is defined. Another recent study of post-high school students attending separate classes within a school collected and compared contact data from sensors, surveys, and online social links [34]. The data include over 300 students wearing sensors for one week and completing a contact diary on one of those days. The data were used to compare capture of contact and patterns with respect to duration and clustering.

We contribute to this growing body of work by estimating contact pairs in a middle school using data from both self-report logs and wireless sensors. The data collection methods are similar to those summarized above so our descriptive summaries can be compared to published findings. The data are useful for modeling transmission of disease and understanding social mixing patterns. The sensor data were used to assess the impact of contact heterogeneity on outputs in a transmission model for influenza [35]. Contacts among pairs of students at the school during the day were recorded by 1) self-report logs only, 2) wireless sensors only, 3) both, or 4) neither. We developed models to estimate the total number of contact pairs, categorized according to physical distance between the pair and presence or absence of verbal communication. Proximal contacts were operationally defined as detected by sensors only, talk/touch contacts detected by self-reported logs only, and proximal talk/touch contacts detected by both sensors and logs. Using these two data sources, we provide descriptive analyses of recorded contacts and estimates of total contact pairs by type of contact. We further describe contact patterns over time and among groups of students. To account for the possibility of incomplete detection, we developed models of the probability of capture to generate estimates of the actual number of each type of contact on the basis of observed data. This serves to highlight the utility of both approaches in describing contact frequency patterns and social mixing among school-aged populations.

## Methods

### Data Collection

#### Study population

We collected contact data from an urban middle school in Utah using wireless proximity sensors and self-report logs on November 28, 2012, a typical mid-week school day. Wireless sensors and self-report logs were distributed to students at the beginning of the school day. The school enrolled 7^th^ and 8^th^ grade students (377 and 301, respectively). The school facility consisted of 26 classrooms in a single-structured, two-floor building. The school schedule has seven class periods and two lunch periods, alternating with 4^th^ period, to accommodate students in the cafeteria.

The study was approved by the University of Utah’s Internal Review Board (IRB _0051285), and the project was also approved under the US Centers for Disease Control and Prevention (CDC) IRB authorization agreement. The school district and school principal approved implementation of the study. Although all students were eligible for participation, teachers, students, and parents were able to opt-out of the study. De-identified datasets used in the analyses presented here are provided in the Supplementary Information (S1–S3 Datasets).

#### Wireless sensors

The wireless sensors used in this study were developed by the Wireless Embedded Sensing Systems Lab (WiESEL) at the University of Utah’s Department of Electrical and Computer Engineering. The wireless ranging enabled nodes [36, 37], or WRENs, send and receive radio signals. The loss (difference) between sending and receiving signal strengths indicated the distance between them. Each WREN contained the Atmel RF233 wireless radio, which used the same frequency spectrum as the 2.4 GHz Wi-Fi radios common in cell phones, laptops, or computers, but used significantly less output power. As a comparison, typical Wi-Fi radios transmit between 10–20 dBm of power. Our current WREN system was set to use -17dBm, which is 1000 times less power based on the logarithmic dB scale. To ensure time synchronization, we sent a clock signal which the WRENs recorded when they started sensing.

The WRENs sent signals approximately every 20 seconds, and recorded continuously. We refer to the record of contact as a ping and assume this represents 20-second contact duration. Aggregation of pings is used to represent duration (e.g., three pings reflect a 1-minute contact). The signal strength for all WRENS was set to record proximities between 0 and 6 feet. The signal could be disrupted by thick clothing, large objects, like shelves or desks, and human bodies, thus the distance range was approximate and contact was considered a binary variable without an estimated distance. Given these properties, the WREN sensors recorded contacts when students’ WREN sensors were 1) approximately facing each other with a clear path between (no obstacles like people or walls), 2) within 6 feet of each other, and 3) in this configuration long enough to record a WREN ping (approximately 20 seconds). These specifications are similar to other recent work [31, 34].

**Fig 1:**
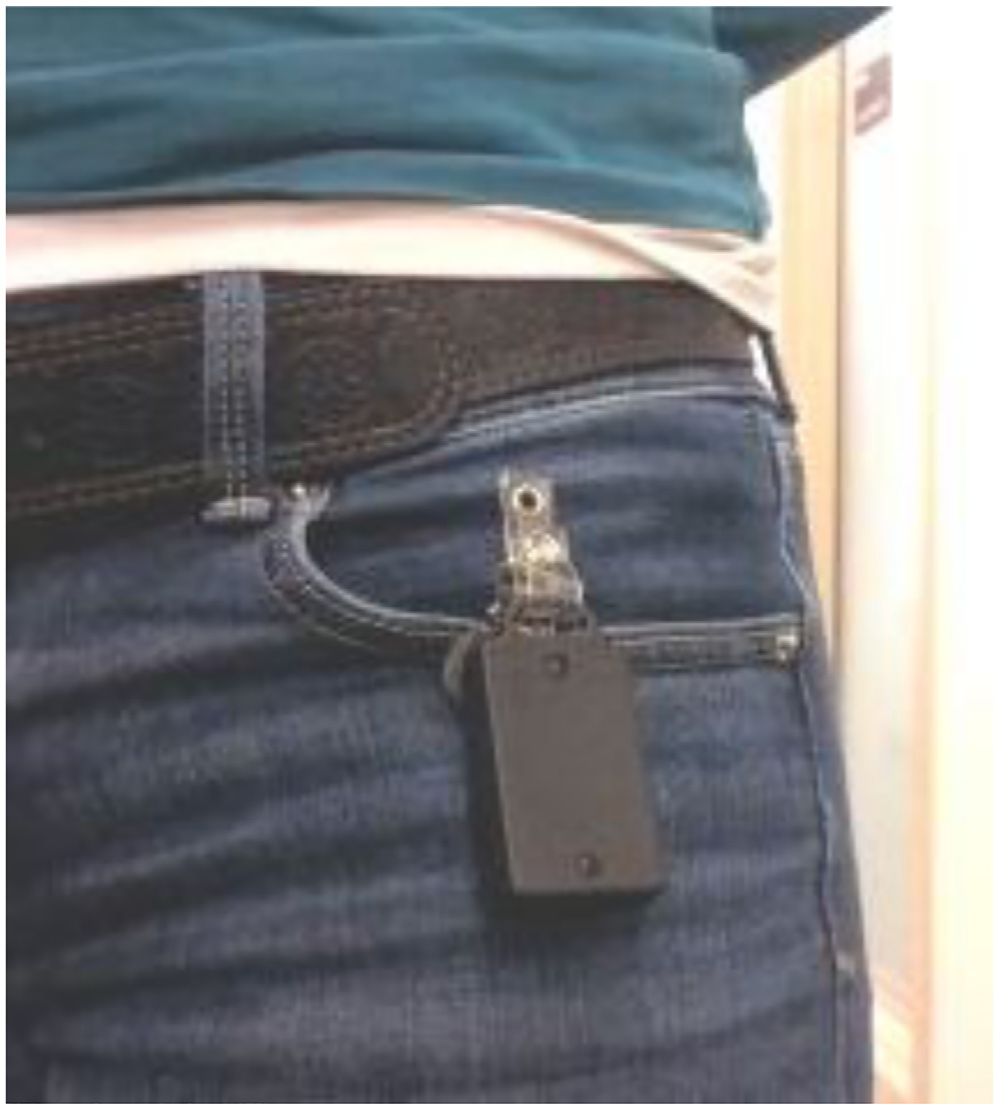
WREN worn by student.

At the time of distribution, students provided the WREN ID (etched into the case) and indicated their gender and grade on a class roster next to their name. The students were instructed to wear the WREN for the entire school day, attached to a clothing item (Fig 1) or in a pocket, in the front. The WREN case was sturdy and water-resistant and could be worn during physical education and while washing hands.

#### Self-report log

The self-report log (hereafter, Log) was distributed in the morning along with the WRENs. Students had the option to participate in both data collection methods, neither, or one or the other. Students providing self-report contact data were encouraged to complete the log throughout, rather than at the end of the study day, in order to improve recall. Students were directed to enter the initials of people they talked to or touched, without regard to the duration. The Log page rows allowed students to record the initials of 56 different persons they talked to or touched, the gender and grade of the person, and the location of contact (i.e., specific class period or during lunch). This space was ample for most, 3.4% of participants listed 56 contacts and 4.1% listed more than 56 using the margins of the page. Logs were collected at the end of the last class period; each completed contact log was assigned a unique Log ID.

The Log ID and WREN ID were linked to a separate study ID (assigned to each student using the school roster).

### Data Processing

As described above, WRENs recorded contacts when participating students’ WRENs were: 1) facing each other with an unobstructed path between, 2) within 6 feet of each other, and 3) in this configuration for at least 20 seconds (1 ping); we defined these as proximal contacts. The Logs recorded information on contacts if students reported talking to each other or having physical contact, defined as talk/touch contacts. If students met both criteria, the contact could have been recorded by both and was termed a proximal talk/touch contact. We assumed no false-positives were recorded by WRENs or reported in Logs.

In addition to the WREN and Log data sets, we generated a Linkable data set to allow matching of WREN recorded contacts to Log reported contacts. This data set included pairwise contacts for individuals who participated in WREN data collection, completed the self-report Log, and had unique signatures. A unique signature was defined as a set of unique initials, gender, and grade within the school. We assumed that this Linkable data set represented a random sample of participants because having a unique signature was not related to participation (Fisher’s Exact test: Logs p = 0.8, WRENs p-value = 0.4) or social contact (Kruskal-Wallis test on degree: Logs p = 0.3, WRENs p = 0.9).

Unique contact pairs were aggregated within time periods defined as class periods, 1^st^ through 7^th^, lunch time, and between classes, and summed to obtain unique time-period specific contact pairs and associated duration. For WREN data, the between-class time period was a sum of contacts from all time segments between classes and lunch.

#### WREN data

We removed extraneous (before and after school) and erroneous data due to midday removal or loss of the sensor (information on these events were recorded by study personnel during the deployment and compared to the sensor data during processing). Raw data were initially recorded as ego (the WREN that recorded contact) and alter (the WREN that sent the signal). Reciprocally recorded contacts were identified in the raw data and used to estimate the probability that a contact was recorded. We removed dually recorded pings occurring between the same two students, with ego/alter reversed, in the same ~20-second window in the WREN data. This process detached the ego and alter designations, although we still used these terms to describe the contact pair. As a result, the consolidated WREN data provided one data record per continuous contact, defined as consecutive ~20-second time windows in which at least one ping was recorded between each pair of students. The duration of a continuous contact was estimated in multiples of 20 seconds. These contact-pair durations were aggregated by time period (class periods, lunch, or between classes). First or second lunch was not specified on the student roster, so we inferred this information from the time-stamped sensor network, which partitioned students into two lunch groups. The few students with contacts in both clusters were assigned to the lunch with the majority of contacts matching that lunch period.

#### Self-report log data

Students reported contacts by their initials, gender, and grade and identified the time period as class period or lunch in the Log. Similar to the WREN data, we used the term ego to identify the student completing the log and alter to identify the listed contact. As a result, the listed contact represented directional contact information. The reported contact’s grade was missing in 1% of reported contacts and the reported contact’s gender was missing in 1%. A set of identical initials, gender, and grade listed as contacts multiple times in a single time period was assumed to represent different students unless that set of initials, gender, and grade was unique to the school (unique signature). Repeated contacts with a unique signature within the same time period were treated as a single contact. Reciprocally reported contacts between unique signature students within a time period were used for assessment but were counted as a single contact pair.

### Data Analysis

Analyses focused on the goal of estimating contact frequency for types of contact (proximal, talk/touch, and proximal talk/touch) and the patterns of contact types through the school day and among groups of students. We will use the term contact pairs (contacts) interchangeably with unique time-period specific contact pairs (contacts) to represent contact between unique pairs of students within a time period.

#### Descriptive analysis

We summarized participation in the Log and WREN portions of the study by gender and grade. Assessment of reciprocal recording and reporting of contact was used to define subgroups for subsequent analyses.

Reciprocal recording of contact by WRENs was calculated using the pre-processed (before excluding double recorded contacts) WREN data. To coincide with the definition for Logs, we defined reciprocal recording for WRENs as contacts occurring in reverse order during the time period. The probability of a WREN recording a contact, *p*_*w*_, was estimated as the number of reciprocal records of contact divided by the total records of contact (duplicates included in both). We hypothesized that reciprocal recording was associated with duration of contact. Thus, reciprocal records and probability of recording were calculated by duration (*d*) of contact ($p_{p}^{d}$). Low reciprocal recording in short duration contacts was used to perform subgroup analyses.

Reciprocal reporting of contact from the Logs was calculated using the Linkable data set. Log reports were reciprocal if the contacts were reported by both students during the same time period (the Log was separated into sections for time period). We estimated the probability of a participant reporting a contact, *p*_*l*_, as the number of reciprocal records of contact divided by the total records of contact (duplicates included in both). We performed sensitivity analysis to determine if the unique signature criterion for the Linkable data set influenced the estimates of reciprocal reporting, and thus probability of capture. There were 319 students in the school with non-unique signatures, represented by 136 sets of initials, grade, and gender. For each unique set, we assigned each student with matching initials, grade, and gender, linked the data sets, and estimated the maximum reciprocal reporting.

We used the Linkable data set to assess the recording overlap for WREN and Log-specific contacts. We performed sensitivity analysis to determine if the unique signature criterion for the Linkable data set influenced the estimates of overlap, analogous to the analysis used for estimating reciprocal reporting. We hypothesized that reciprocal reporting was associated with duration of contact and therefore recording overlap for WREN and Log. Thus, reciprocal reports and probability of reporting were calculated separately for contacts reported by WREN and Log, by duration of contact ($p_{l*}^{d}$).

Unique time-period specific contact pairs from both data sources were analyzed to describe social mixing patterns across time periods and by gender and grade. Mean and median contact degree were calculated as the average and median total number of unique contacts per student for each time period.

We described the duration of WREN contacts graphically, and used Wilcoxon non-parametric tests to compare contact duration by time period, gender, and grade.

We created contact matrices using the mean and median number of unique time-period specific contacts per person per day by gender and grade. Contact pairs were counted twice if the pair included contacts from the same gender or same grade; if the pair included students from a different gender or grade, then the contact was counted for each order of the contact. The contact matrices from WREN data used all contact pairs and those with a total duration 20 seconds.

Assortativity, the tendency to socially mix with others in the same grade or gender was assessed graphically and based on the assortativity coefficient [38]. Permutation was used to test whether coefficients were different than would be expected from a random network. Each value was compared to a distribution of values from 1000 random networks. The random networks were based on random re-assignment of edges performed by 1) retaining the degree distributions within groups and keeping the group membership of the nodes and 2) randomly assigning group membership and retaining the same overall degree distribution.

#### Estimates of contact pairs

**Fig 2:**
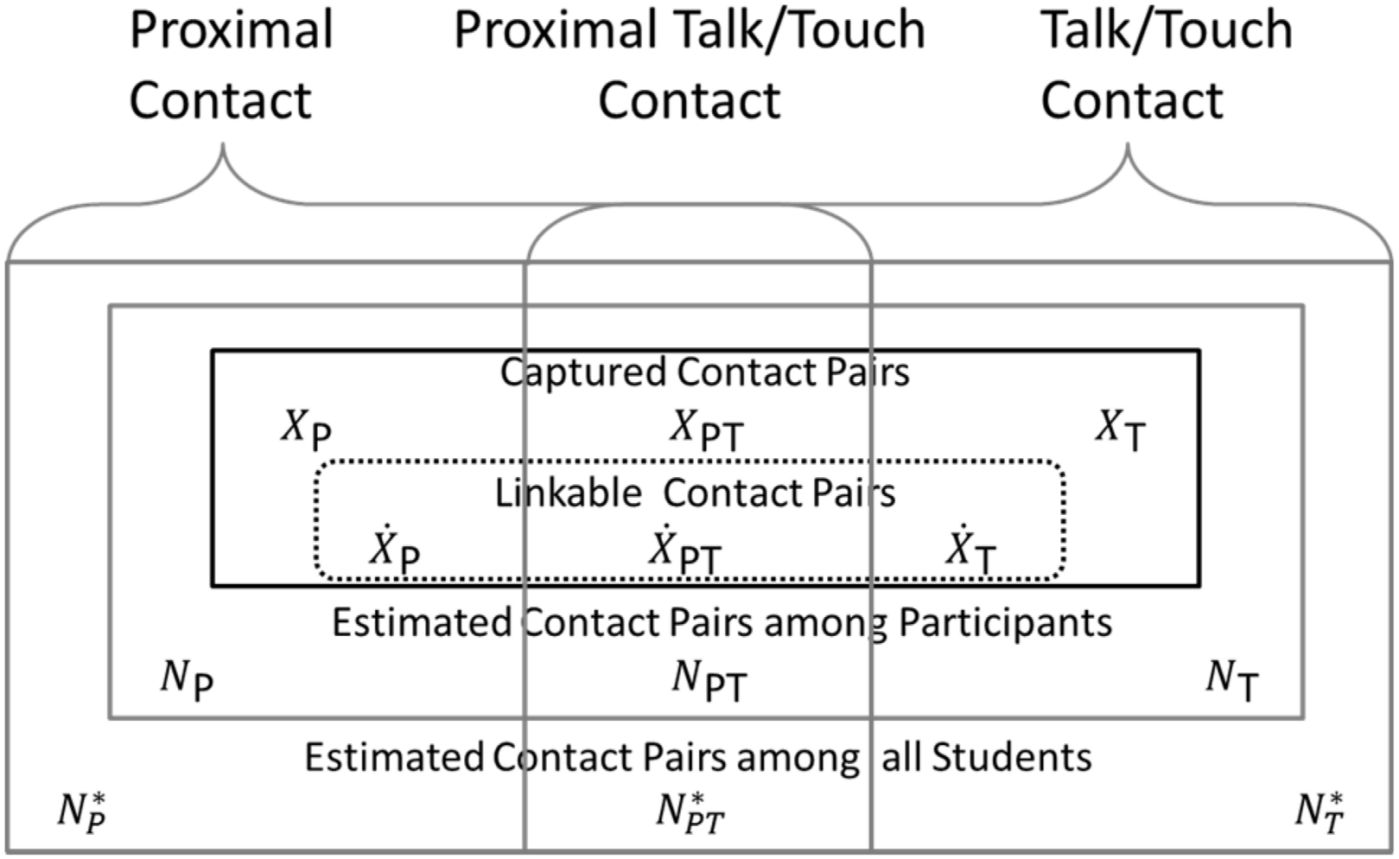
Framework and notation for enumeration of contact pairs. Contact pairs are either proximal only, proximal talk/touch, or talk/touch only. The linkable data set is a subset of the captured contact pairs. We observe the quantity (*X*_P_ + *X*_PT_)as the total WREN contacts and (*X*_T_ + *X*_PT_) as the total Log contacts.

Contact pairs recorded by WREN or Log were defined as observed contact pairs (X); the subset with unique signatures were defined as Linkable contact pairs ($\dot{X}$) (Fig 2). Contacts recorded by the WRENs are proximal (P), by self-report Logs are talk/touch (T), and by both are proximal and talk/touch (PT) (Fig 2). We observed the quantity, (*X*_P_ + *X*_PT_), where $X_{P}=\Sigma_{d}X_{P}^{d}$ and $X_{PT}=\Sigma_{d}X_{PT}^{d},$ as the total WREN recorded contact pairs summed across durations, and (*X*_T_ + *X*_PT_) as the total Log recorded contact pairs, but did not observe the separate quantities *X*_P_, *X*_PT_, or *X*_T_ directly. To estimate the number of observed pairs that were proximal and talk/touch (*X*_PT_), we applied the proportion observed for the Linkable pairs $\left(\frac{{\dot{X}}_{\text{PT}}}{\left({\dot{X}}_{\text{T}}+{\dot{X}}_{\text{PT}}\right)+\left({\dot{X}}_{\text{P}}+{\dot{X}}_{\text{PT}}\right)}\right)$ to the all WREN (*X*_P_ + *X*_PT_) and Log (*X*_T_ + *X*_PT_) recorded pairs as shown in Eq 1 below, applying the proportion of contact by duration based on Linked data.

The following three subsections detail the estimated probability of capture, participation, and estimation of contact pairs. The probability of capture, based on reciprocals, was used to estimate all contact pairs (recorded and unrecorded) among participants from observed contact pairs (*X*_P_, *X*_T_, *X*_PT_). Projection to estimated contact pairs among all students was based on participation rates in the study.

#### Probability of capture

The probability of capture was based on the reciprocal recording within each source, Log and WREN, similar to methods in [33]. Differences in reciprocal recording of WRENs by duration of contact were incorporated into the estimation procedure. The reciprocal reporting in Logs was higher for contacts that were also recorded by WRENs (Fisher’s exact test p<0.0001). Thus the probability of a participant reporting a contact was estimated separately for Log contacts also recorded by WREN ($p_{l}^{*}$). The probability of capture for each type of contact was estimated from $p_{w}^{d}$, *p*_*l*_, & $p_{l}^{*}$ (Table 1). These equations reflect the fact that uncaptured contacts occurred only if *both* students failed to record it.

**Table 1:** Notation and calculations for probability of capture for proximal, talk/touch, and proximal talk/touch contact pairs.

	Probability that single student records	Probability that contact is captured
**Proximal Contact Pair**	$p_{w}^{d}$	$p_{p}^{d}=1-{\left(1-p_{w}^{d}\right)}^{2}$
**Talk/Touch Contact Pair**	*p*_*l*_	*p*_T_ = 1 − (1 − *p*_*l*_)^2^
**Proximal Talk/Touch Contact Pair**	$p_{w}^{d}p_{l}^{*}$	$p_{\text{PT}}=\left[1-{\left(1-p_{w}^{d}\right)}^{2}\right]\left[1-{\left(1-p_{l}^{*}\right)}^{2}\right]$

#### Participation

Although participants were included in the study all day, actual participation data were assessed by time period, gender, and grade to assess whether actual participation differences that could affect estimation occurred. The number of contacts recorded from the Logs and WREN was compared across time periods. The participation fraction used to estimate total contacts was the average participation across time periods. Time-period specific participation fraction was constant but less than the participation in the study overall.

Estimated contact pairs among all students were calculated as the estimated contact pairs (recorded and unrecorded) among participants divided by the proportion of contact pairs possibly captured for each type (*π*_*T*_, *π*_*P*_, *π*_*PT*_). The proportion of contact pairs possibly captured was estimated as participation fraction squared for WRENs and 1 minus the non-participation fraction squared for Logs, because participants could report contact with non-participants [39]. For PT contacts, we estimated the proportion of contact pairs possibly captured as the product of the proportion of contact pairs possibly captured for Log and for WREN.

#### Estimation

We assumed that contact pairs among participants for each type of contact (P, T, PT) were binomial with specified total contact pairs among participants (*N*) and probability of capture (*p*). The probability of capture, *p*, for P was estimated by duration of contact, for T was estimated from the full data set, and for PT estimated based on the proportion observed in the Linkable data set, $\dot{X}$:

$$X_{P}^{d}\sim binom\left(N_{pP}^{d},p_{P}^{d}\right)$$

$$X_{\text{T}}\sim binom\left(N_{\text{T}},p_{\text{T}}\right)$$

$$X_{PT}^{d}\sim binom\left(N_{PT}^{d},p_{PT}^{d}\right),$$ (1)

where

$X_{PT}^{d}=\frac{{\dot{X}}_{PT}^{d}}{\sum_{d}{\dot{X}}_{PT}^{d}}\left[\left(X_{\text{P}}+X_{\text{PT}}\right)+\left(X_{\text{T}}+X_{\text{PT}}\right)\right]\frac{{\dot{X}}_{\text{PT}}}{\left({\dot{X}}_{\text{T}}+{\dot{X}}_{\text{PT}}\right)+\left({\dot{X}}_{\text{P}}+{\dot{X}}_{\text{PT}}\right)}$, and the quantities, (*X*_P_ + *X*_PT_) and (*X*_T_ + *X*_PT_), are recorded total contacts from WRENs and Log, respectively.

The estimates of contact pairs among participants were calculated using duration-specific probability of capture and number of contact pairs for P and PT as:

$$N_{P}^{d}=X_{P}^{d}/\left(p_{P}^{d}\right)$$

$$N_{\text{T}}=X_{\text{T}}/\left(p_{\text{T}}\right)$$

$$N_{PT}^{d}=X_{PT}^{d}/\left(p_{PT}^{d}\right)$$

Projection of estimated contact pairs among participants to contact pairs among all students was calculated as:

$$N_{P}^{*}=\Sigma_{d}N_{P}^{d}/\pi_{P}N_{T}^{*}=N_{\text{T}}/\pi_{T}$$

$$N_{PT}^{*}=\Sigma_{d}N_{PT}^{d}/\pi_{PT}.$$

Variance was determined via the Delta method for 95% confidence intervals.

## Results

### Descriptive Analysis

There were 678 students at school on the day of the study. Almost all students (630, 93%) wore a WREN that recorded data, and the majority of students (446, 66%) also completed a self-report Log. There were 238 (35%) students who completed a Log who had a unique set of initials, grade, and gender and, therefore, linkable to assess reciprocal reporting and overlap with WREN. The distribution of participation across gender and grade was similar for WREN, Log, and Linkable data sets (Table 2).

**Table 2:** Grade and gender of school population and participants: number of students and percent of total.

Grade	Gender	Students Present at School (%)	Students who Wore WREN (%)	Students who Completed Log (%)	Students with Unique Signature who Completed Log (%)
**7**^**th**^	**F**	192 (28%)	176 (28%)	133 (30%)	69 (29%)
**7**^**th**^	**M**	185 (27%)	178 (28%)	112 (25%)	59 (25%)
**8**^**th**^	**F**	155 (23%)	142 (23%)	117 (26%)	62 (26%)
**8**^**th**^	**M**	146 (22%)	134 (21%)	89 (20%)	52 (22%)
**Total**		678	630	446	238

During processing 40,056 WREN records were matched, representing reciprocal recording of a contact pair. Reciprocal recording in WREN depended on contact duration. Reciprocal recording was low (16%) for the 16,387 contact pairs of 20 second duration during class time and lunch. For longer duration contacts, reciprocal recording was high, 69% for 40 seconds and 84% for 1 minute, and approximately 94% for contacts longer than 1 minute.

Reciprocal reporting in Logs, based on 1,575 contacts between unique signatures only, was just 30%. There was little sensitivity in reciprocal reporting of contacts in a Log by matching students with non-unique signatures. From the 5,285 possibly linked contact pairs, 28% were reciprocally reported.

**Fig 3:**
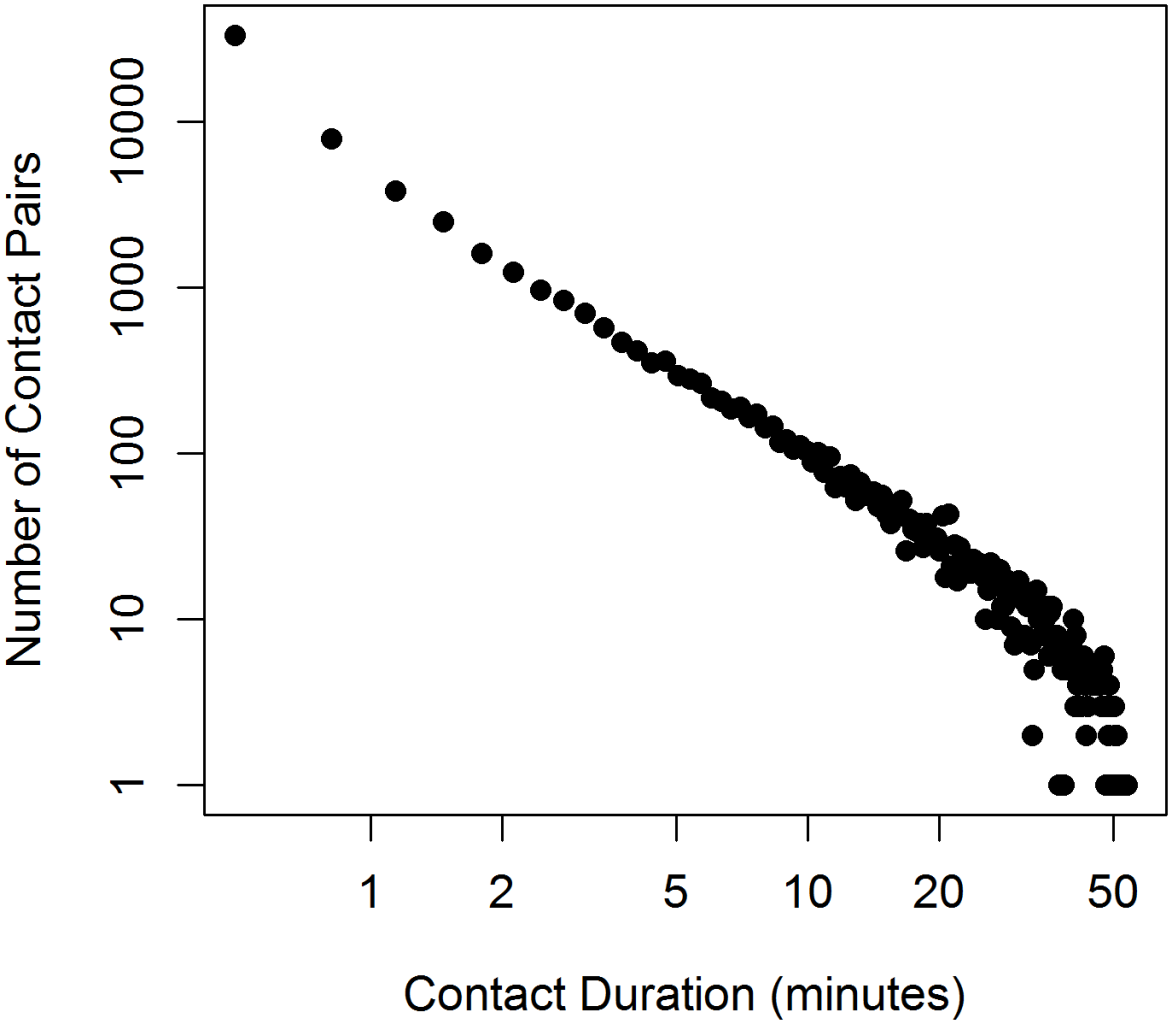
Frequency of contact pairs by contact duration (in minutes) on a log-log scale.

WRENs recorded 60,292 contact pairs representing any proximal contact between two students and 27,101(45%) contact pairs with duration more than 20 seconds, as defined by at least two consecutively recorded pings (ping being a recorded signal within a 20-second window), aggregated by time periods. Of the contacts at least 20 seconds, 52% of contacts were between 20 seconds and 1 minute (inclusive) and 19% were more than 5 minutes in duration (Fig 3). The Logs recorded 10,484 contact pairs representing talk/touch contact between two students.

**Fig 4:**
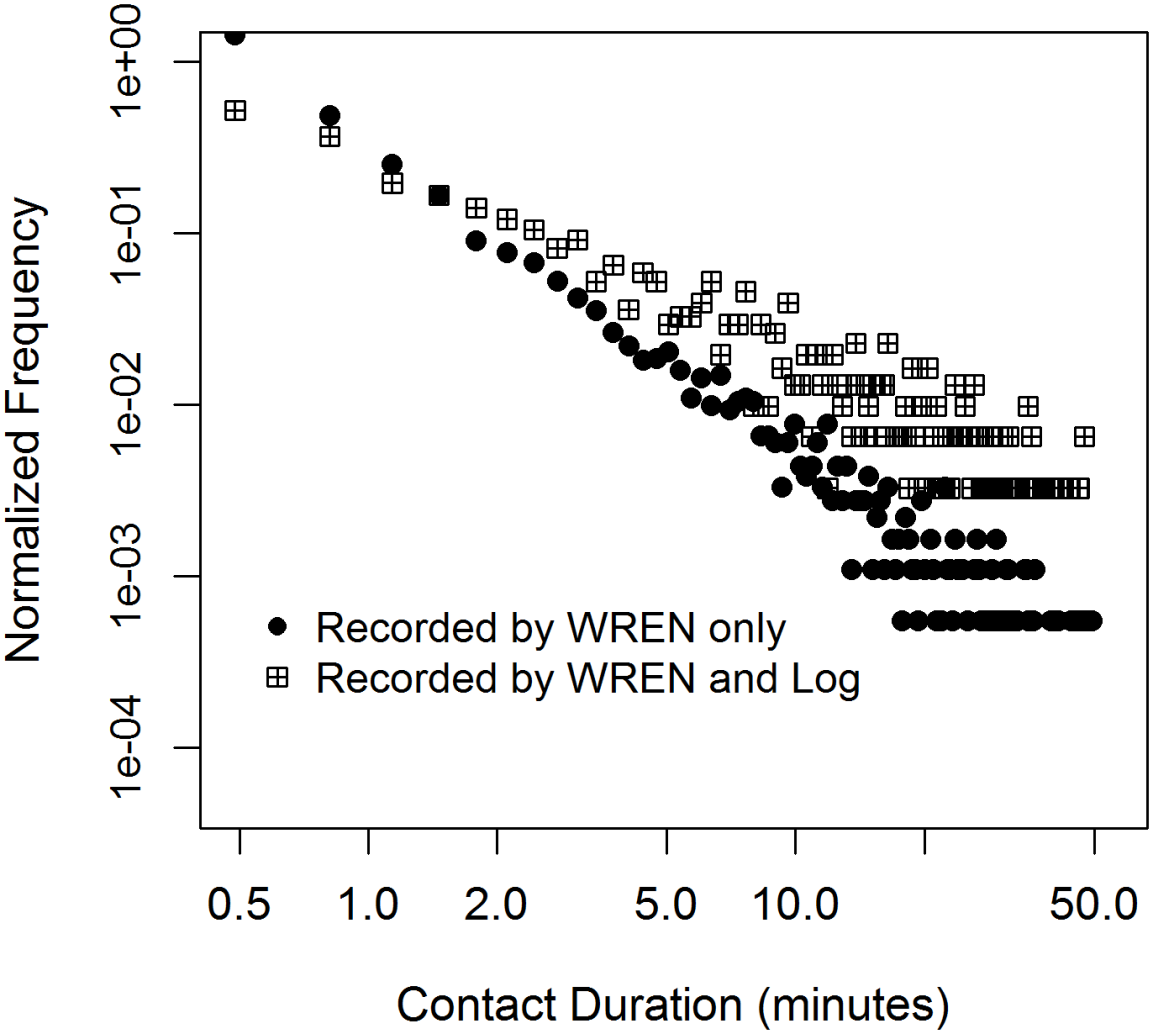
Normalized frequency of contact pairs by contact duration (in minutes) on a log-log scale for contacts captured by WREN only and contacts captured by both WREN and Log.

We recorded 6,303 contact pairs between participating students with unique signatures during class-time and lunch; 864 of these (14%) were recorded by both WREN and Log, 711 (11%) by Log only, and 5,592 (75%) by WREN only. Overall, 55% of the Log-reported contact pairs were also recorded by WRENs, and 15% of the WREN-recorded contact pairs were also reported in the Logs. There was little sensitivity in the estimates of overlap between WREN and Log to linking on unique signatures only. From the 20,207 possibly linked contact pairs, 10% were recorded by both WREN and Log, 10% by Log only, and 80% by WREN only. The durations of contact pairs recorded in both WRENs and Logs were longer than durations of contact pairs recorded in WRENs only (Fig 4, mean: 6.3 vs 2.4, median: 2.28 vs 0.65).

Reciprocal recording by WREN of contacts recorded by both WREN and Log was almost exactly the same as reciprocal recording by WREN overall, for the duration categories used above. Reciprocal reporting by Log of contacts recorded by both WREN and Log was higher (41%) than the overall Log reciprocal reporting.

The number of WREN and Log contact pairs varied by time period during the school day (Table 3). Contact was fairly consistent across the class periods, summarized by number of contact pairs and mean degree per student. Slightly higher reported contact during first period was likely due to enthusiasm of participants at the start of the study. The degree was higher for both Log and WREN during lunch than during class periods.

**Table 3:** Summary of captured contact pairs from Log and WREN during each period of the school day and overall.

	Time Period									
	1	2	3	4	5	6	7	Lunch	Between Classes^4^	All Day
**Log**										
**Participants**^1^	393	381	357	326	313	285	290	337	-	446
**Contacts**	1680	1393	1367	1181	1112	931	1182	1638	-	10,484
** % of Total**	16	13	13	11	11	9	11	16	-	
**Mean Degree**	4.2	3.6	3.8	3.6	3.5	3.2	4.0	4.8	-	23.2
**Median Degree**	3	3	3	3	3	3	3	4	-	22
**Degree IQR**	2–5	2–5	2–5	2–5	2–5	2–4	2–5	2–6	-	13–31
**WREN**										
**Participants**^2^	606	622	625	627	621	620	615	629	630	630
**Contacts**	3637	4226	4420	4062	4109	3818	3631	12,099	20,300	60,292
** % of Total**	6	7	7	7	7	6	6	20	34	
**Mean Degree**	12.0	13.6	14.1	13.0	13.2	12.3	11.8	38.5	57.4	133.1
**Median Degree**	11	12	13	12	11	12	10	37	57	133
**Degree IQR**	7–16	7–18	8–18	8–18	7–17	8–16	7–15	27–49	44–70	111–157
**Mean Duration**^3^	2.9	3.3	3.3	3.4	3.3	3.5	2.8	1.1	0.5	1.9
**Median Duration**^3^	0.7	1.0	1.0	1.0	1.0	1.0	0.7	0.3	0.3	0.3
**Duration**^3^ **IQR**	0.3–2.6	0.3–2.6	0.3–2.9	0.3–2.9	0.3–2.9	0.3–2.9	0.3–2.6	0.3–1.0	0.3–0.3	0.3–1.3
**WREN >20 seconds**										
**Participants**^2^	600	617	616	620	611	613	598	622	625	630
**Contacts**	2384	2835	2868	2739	2790	2545	2323	4844	3773	27,101
** % of Total**	8.8	10.5	10.6	10.1	10.3	9.4	8.6	17.9	13.9	
**Mean Degree**	7.9	9.2	9.3	8.8	9.1	8.3	7.8	15.6	10.8	64.8
**Median Degree**	7	8	9	8	8	8	6	15	11	64
**Degree IQR**	5–10	5–12	5–13	5–12	5–12	4–11	4–9	10–20	7–14	51–79
**Mean Duration**^3^	4.2	4.7	4.9	4.9	7.8	5.0	4.3	2.3	1.2	3.8
**Median Duration**^3^	1.6	1.6	2.0	1.6	2.0	2.0	1.6	1.3	0.7	1.3
**Duration**^3^ **IQR**	1.0–4.6	1.0–4.6	1.0–5.2	1.0–4.9	1.0–4.9	1.0–5.2	1.0–4.6	0.7–2.6	0.7–1.0	0.7–3.6

The total Log contacts were 10,484 from 446 students and the total WREN contacts were 60,292 from 630 students.

^1^ Those who reported contact during the time period.

^2^ Those who had a recorded contact during the time period.

^3^ Duration in minutes. Lower values for 1^st^ and 7^st^ period likely because of distribution and collection of WRENs.

^4^ Between class contacts were only recorded by WRENs, not logs.

Mean duration of contact significantly differed among class-time, lunch, and between classes (ANOVA p-value < 0.0001, Table 3), using all WREN data or any duration threshold. Duration of contacts (more than 20 seconds) by gender groups differed only slightly for girl-girl (3.8 min), boy-boy (3.6 min), and girl-boy (3.9 min). Duration of within-grade contacts (more than 20 seconds) did not differ for 7^th^ and 8^th^ graders (4.0 min for both), but between-grade contacts were significantly shorter (2.7 min, Wilcoxon p-values < 0.0001).

**Fig 5:**
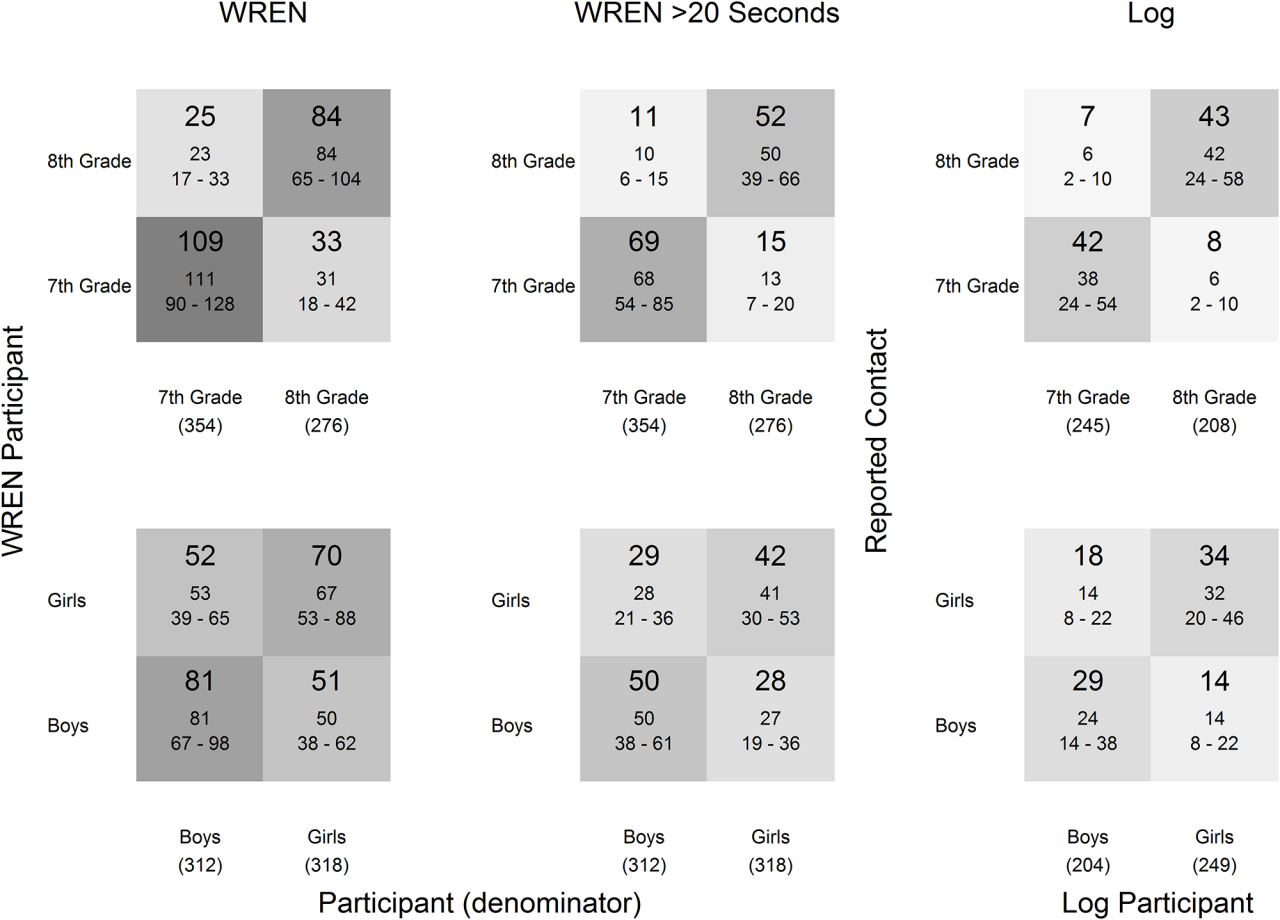
Contact matrices from observed WREN-recorded contacts (all durations and > 20 seconds) and Log-reported contacts using data from class period and lunch. The total contacts recorded between two groups are divided by the number of participants in the column group (given in parentheses) to provide an average number of contacts. Numbers per cell are mean (top), median, and interquartile range (25th and 75th percentile).

The mean (and median, which were similar) number of contacts per student per day varied among WREN, WREN >20 seconds, and Log (Fig 5). Although the patterns of contact between groups appeared to be similar among data sets, the magnitude of the assortativity coefficients for grade, gender, and degree differed somewhat by data source and time period (Table 4). Both grade and gender assortativity were higher using Log data than using WREN data, for class-time and lunch. Grade assortativity was prominent in each data set but stronger during class-time. The gender and grade assortativity using WREN data were higher for both lunch and between classes when the 20-second contacts were removed. Because most classes were grade-specific, the high grade assortativity was assumed to be a result of school schedule, not student behavior. Grade assortativity from the WREN data decreased during lunch compared to class-time but was approximately the same for Log data. The gender assortativity was higher during lunch compared to class-time. Degree assortativity was low overall. Although the magnitudes varied, all assortativity coefficients were at least four times the maximum calculated from the set of 1000 random networks, indicating non-random mixing. An upper bound on assortativity coefficients, given the group sizes and degree distributions, was 1 if maximum assortative mixing had occurred.

**Table 4:** Assortativity coefficients by data source for class period, lunch and between classes.

Data Source	Class Only			Lunch Only			Between Class Only		
	Grade	Gender	Degree	Grade	Gender	Degree	Grade	Gender	Degree
**All WREN**	0.67	0.16	0.10	0.23	0.25	0.05	0.36	0.17	0.06
**>20seconds WREN**	0.71	0.18	0.12	0.40	0.45	0.14	0.67	0.41	0.06
**Log**	0.80	0.31	0.09	0.78	0.64	0.15	-	-	-

WREN data were used including all contacts and also only those greater than 20 seconds. Between class contacts were only recorded by WREN.

### Estimates of Contact Pairs

#### Probability of capture

Probability of capture by WREN for proximal contacts was calculated for contact pairs of 20 seconds (0.29), 40 seconds (0.9), and 1+ minutes (0.99) (Table 5). The probability of capture by WREN of proximal talk/touch contacts was almost identical for the duration categories. The probability of capture by Log of proximal talk/touch contacts was 0.65. Thus, the probability of capturing a proximal talk/touch contact was estimated as 0.19, 0.59, and 0.65 for contact pairs of 20, 40, and 60+ seconds, respectively.

**Table 5:** Measured contact characteristics between pairs who participated in WREN or Log portions of study, based on class-time and lunch.

Variable	Description	Data Value or Estimate
***X***_**P**_ + ***X***_**PT**_	Total Observed WREN contacts	39,992
***X***_**T**_ + ***X***_**PT**_	Total Observed Log contacts	10,484
***X***_**PT**_	Estimated Overlap WREN/Log contacts	5,949
**${\dot{X}}_{P}$**	Observed WREN-only (Linkable data set)	5,592
**${\dot{X}}_{T}$**	Observed Log-only (Linkable data set)	711
**${\dot{X}}_{PT}$**	Observed WREN and Log (Linkable data set)	864
***p***_***w***_	Probability of a WREN recording a contact (20, 40, 60+ seconds)	0.16, 0.69, 0.92
***p***_***l***_	Probability of a Log reporting a contact	0.30
**$p_{l}^{*}$**	Probability of a Log reporting a contact (Linked data set)	0.41
***p***_**P**_	Probability of capturing a proximal contact (20, 40, 60+ seconds)	0.29, 0.9, 0.99
***p***_**T**_	Probability of capturing a talk/touch contact	0.51
***p***_**PT**_	Probability of capturing a proximal talk/touch contact (20, 40, 60+ seconds)	0.19, 0.59, 0.65

The probability of capturing a talk/touch contact during class time or lunch by at least one student’s Log was 0.51.

Reciprocal reporting and therefore probability of capture varied somewhat by class period and to a lesser degree by gender and grade. The differences were small and final estimates were not sensitive to these small differences so they were not incorporated.

#### Participation

The participation fraction for WRENs was 0.93 so the estimated proportion of the P contact pairs possibly captured was *π*_*P*_ = 0.93^2^ = 0.8649. The average time-period specific participation fraction for Logs was 0.5, so the estimated proportion of the T contact pairs possibly captured was *π*_*T*_ = 1 –(1–0.5)^2^ = 0.75. The estimated proportion of the PT contact pairs possibly captured was *π*_*PT*_ = 0.75 x 0.93^2^ = 0.6487.

#### Estimation of total contact pairs

Using the overlap, probability of capture, and study participation rates, we estimated 87,060 total and 24,254 longer than 20 seconds proximal-only contact pairs, 11,535 talk/touch-only contact pairs, and 24,293 proximal talk/touch contact pairs occurred during class-time and lunch (Table 6). Each student had an estimated 363 total contacts and 177 if we discount the very short duration proximal contacts.

**Table 6:** Estimated class-time and lunch contact pairs based on duration-specific probability of capture.

	Estimated Participant Contact Pairs Adjusted for Imperfect Capture	Estimated Total Contact Pairs (95% confidence interval)
**Talk/Touch**	*N*_T_ = 8,651	$N_{T}^{*}=11,535\left(9,331-13,739\right)$
**Proximal Talk/Touch**	*N*_PT_ = 13,630	$N_{PT}^{*}=24,293\left(21,668-26,914\right)$
**Proximal**	*N*_P_ = 75,298	$N_{P}^{*}=87,060\left(85,045-89,079\right)$
**Proximal longer than 20 seconds**	$N_{P}^{>20}=20,977$	$N_{P}^{*>20}=24,254\left(23,496-25,014\right)$

**Fig 6:**
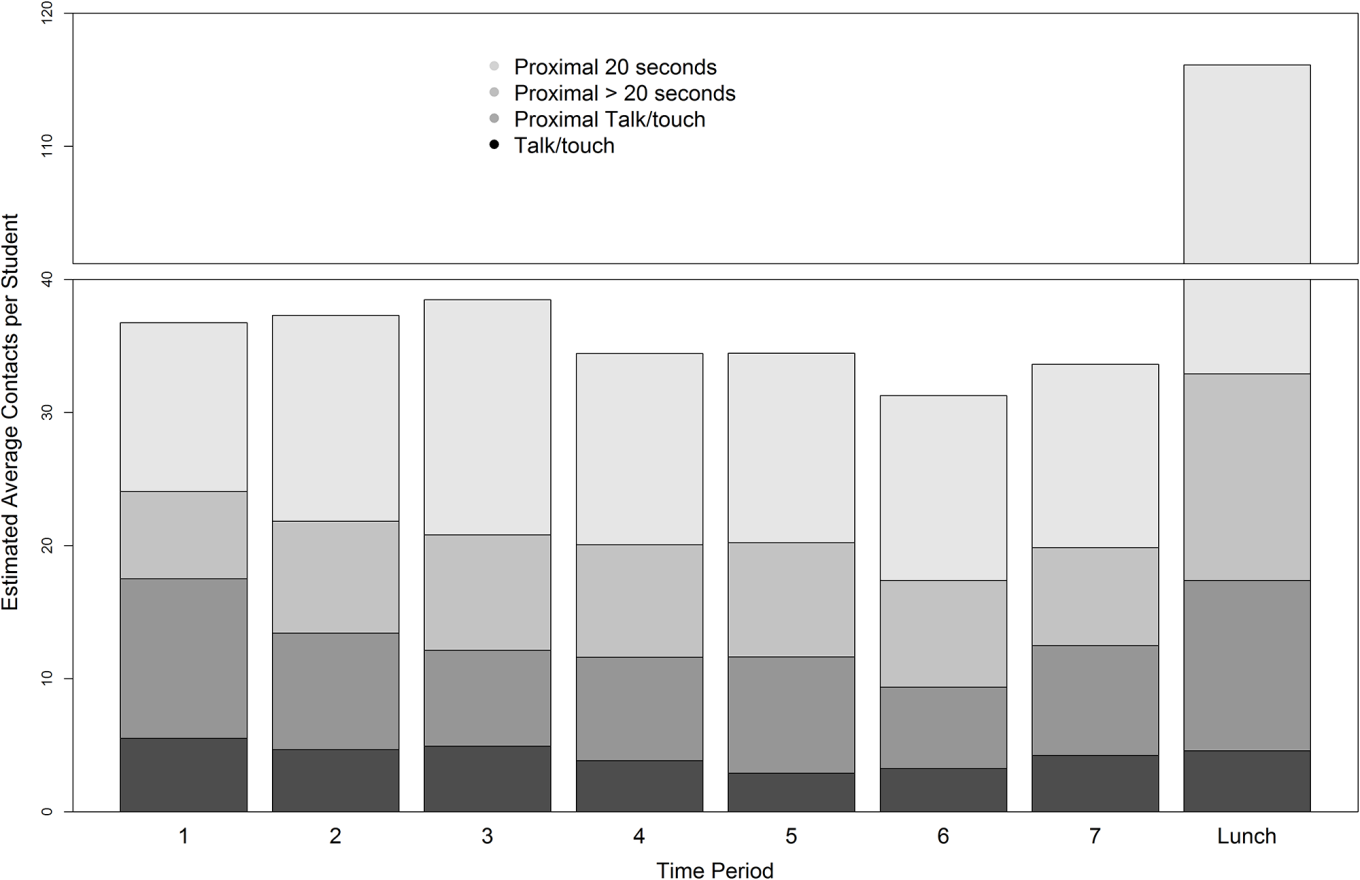
Estimated average T, PT, and P (separated for ≤ and > 20 seconds) contacts per student during class periods and lunch.

The estimated T, PT, and P contacts per student were similar across class periods but different during lunch (Fig 6). The proximal only contacts were mostly of very short duration and dominated lunch, although on average 43% of class-time contacts were also very short. During class-time, PT and P>20 (proximal > 20 seconds) contacts per student were about the same. T only contacts were few and occured in situations, for example, when two people talked at a distance or when they talked without their bodies facing each other (WRENs did not have unobstructed path).

**Fig 7:**
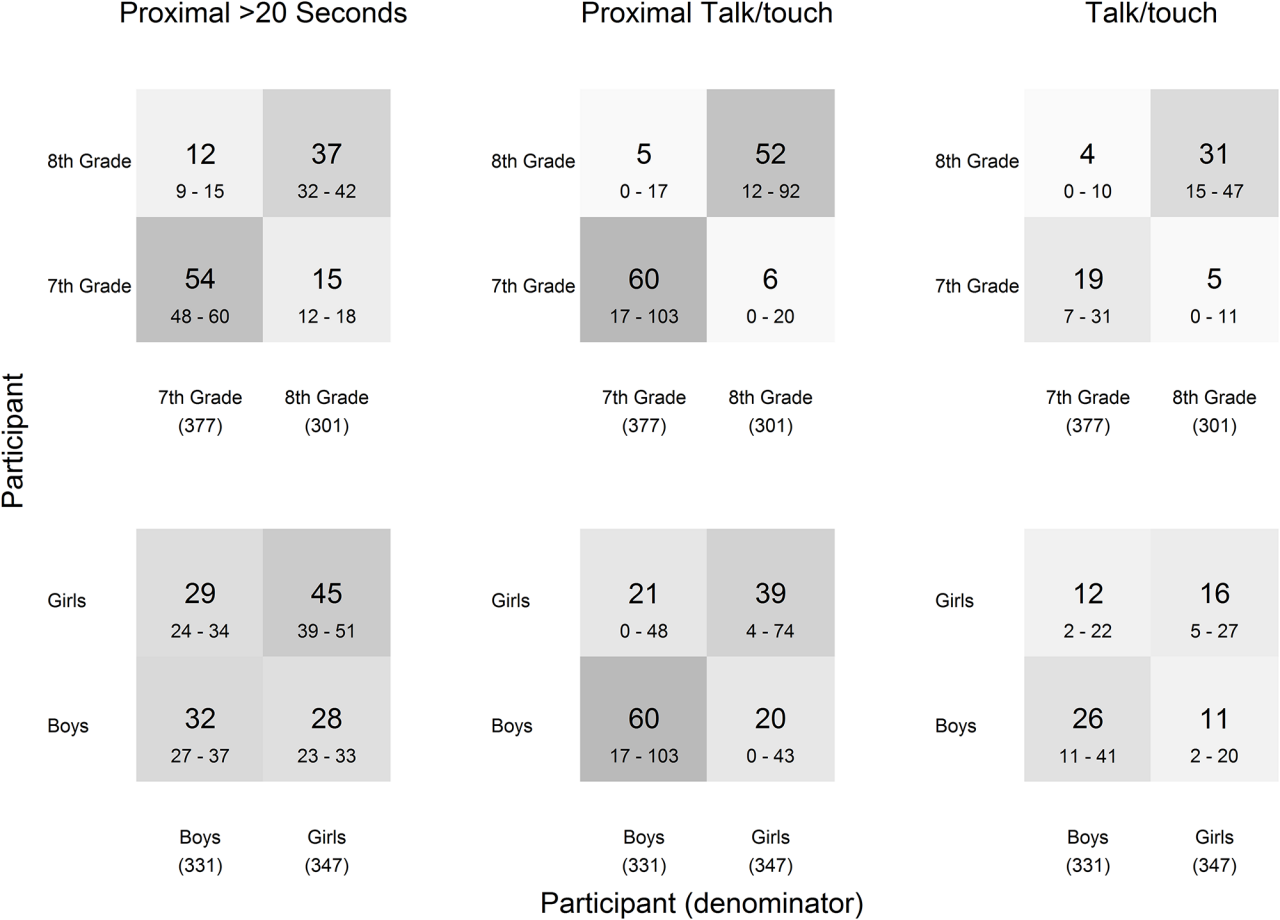
Contact matrices of estimated average contacts per student. Contacts matrices are presented for P (>20 seconds), PT, and T contacts per student during class-time and lunch and include 95% confidence intervals (below).

There were differences in grade and gender assortativity by the type of contact (Fig 7). There were fewer T contacts per student overall, but more were reported between 8^th^ graders than between other groups. The assortativity, by both grade and gender, were higher for PT contacts. P contacts, shown here estimating only contacts greater than 20 seconds, had moderate grade assortativity and little gender assortativity. The contact matrices incorporating all duration P contacts had similar assortativity, but two to three times the number of contacts per student.

## Discussion

We describe contacts and mixing among students in a 678-student urban middle school for one day based on data from a self-report log and wireless sensors. Contact frequency and patterns of mixing differ between data sources and vary among time periods. Class-time contacts are, on average, twice the duration of lunch and four times the duration of between-class contacts. Unique contact pairs per time period depend on data source; WREN degree during lunch or between classes is at least twice the degree during class-time but Log degree is only 30% higher during lunch than class-time. Assortativity measures from Log data are higher than WREN data, overall. Grade assortativity in WREN data can be up to two times higher during class-time than during lunch or between classes. Gender assortativity is approximately 50% higher during lunch than class-time for both data sources. High grade assortativity and relatively low gender assortativity was also seen in French high school data [22]. We report estimates of the total number of contact pairs separated by definitions of talk/touch, proximal talk/touch, and proximal that account for imperfect capture and incomplete participation. Estimated proximal contacts longer than 20 seconds during class-time and lunch occurred at approximately the same frequency as proximal talk/touch and at 2 fold higher frequency than talk/touch contacts. Total unique time-period specific contacts longer than 20 seconds per student per day were estimated at approximately 145. The data and estimates reported here can be used for modeling disease transmission and understanding social interactions and to improve understanding of the similarities and differences between self-report and sensor methods for collecting data on contact.

As hypothesized, sensor data have higher degree (approximately three times) and frequency of contact (two to seven times) than self-report data. This compares well with one study; [34] sensor degree was three times self-report degree and frequency was six times. Another study [33] found sensor degree to be in the range of ten times the self-report degree and frequency 22 times. Their findings applying a duration threshold of 5 minutes were more similar to ours, sensor degree three times self-report and frequency four times. The differences in our data are due in part to Log data (by definition) recording contacts involving touch/talk and WRENs recording proximal contact information. Although touch contact requires proximity, a conversation could occur over a distance, or without students’ WRENs facing each other, or happen with an obstacle between students. Also students could be in proximity for a long time without talking or touching. The differences are also due in part to the differing capture probability of the two methods.

We estimate that 50% of contacts meeting the talk/touch definition were reported in Logs, based on the reciprocal reporting between pairs of students. The reciprocal reporting in Logs (30%) was slightly higher than a recent study (23.5%) with very similar methods and environment but lower participation [33], but substantially lower than another recent study with similar methods and environment (62%) [34], a study that asked primary school students to report who they spend most time with (61%) [3], and a study with adults (65%) [27]. Low matching of reported contacts in our study could have been due to: 1) incorrectly reported initials or 2) incorrect time period reporting. Initials could have been reported incorrectly; our analyses above indicate reciprocal reporting estimates were not sensitive to assigning possible matches of unique signatures. When we allow matching reported contacts any time during the day (not just during the same time period) there were only an additional 4% of contacts reciprocally reported. Thus, we assume low recirpocal reporting was not due to matching errors in the data but simply low reporting of contacts by students. In contrast, we estimate that 96% of contacts meeting the proximal longer than 20 second duration contact definition were recorded by the WRENs.

The Logs and WRENs have different capture probabilities and also target different definitions of contact. Approximately half of Log reported contacts were also recorded by WRENs but only 15% of WREN recorded contacts were also reported in a Log. The percent of self-reported contacts that were recorded by sensors is lower than reports from other studies, [33] reports 85% and [34] reports 70%. The percent of sensor contacts that were also reported in Logs was much lower (4%) in [33] and much higher (41%) in [34]. There are at least three reasons for contacts to be recorded only by one method: 1) there were different sampling frames, 2) one method missed capturing the contact, and 3) the definition of contact was different. We acknowledge that the sampling frame was different, but we used a subset that participated in both and could be identified (and performed sensitivity analysis). The probability of capture differs, as discussed above. The definition of contact differs for the methods and is the basis for our estimating three types of contact. Based on methods described in [33], applied to contacts longer than 20 seconds in duration, we estimate that 86% percent of the Log-only contacts were not recorded by WREN because of a difference in the definition in contact (not due to under-recording of WRENs). We similarly estimate that 59% of WREN-only contacts were not reported in Logs because of a difference in the definition of contact (not due to under-reporting in Logs). This compares well to their [33] estimate that approximately 60% of sensor-only contacts were not reported in the survey because of a difference in the definition of contact.

Comparison of contact estimates with other studies is approximate due to differences in many factors (population size, design, and definition of contact) including participation rate. Non-participation in collection of contact data affects both sensors and self-report, albeit somewhat differently because self-report can include non-participants. Summaries of contact degree and frequency underestimate true values, while summaries of assortativity, overlap, and reciprocal capture could under- or over-estimate true values. Participation in our study was very high for sensors (93%) and 72% of these participating students also completed a log (possibly hindered by the perceived burden). Participation was similar for both methods across grade and gender. Two similar studies in high schools had lower participation; one study with 86% sensor participation and approximately 40% of those completing at least one of two surveys [34] and one study with much lower participation, 50% with sensors and 26% of those also completing contact surveys [33]. Differences in findings among recent studies could be partially due to differences in participation.

The difference in assortativity among contact types could also be important for understanding disease transmission. Estimated proximal talk/touch contacts were two to ten times more common between students of the same grade or same gender than between students of different grade or different gender but for estimated proximal contacts they were less than three times more common, and much less for gender. Proximal talk/touch contacts likely have greater intimacy than proximal contacts and we hypothesize that they are associated with higher transmission probability. The greater number of proximal contacts across gender and grade groups could be the transmission route that links clusters and allows wider spread of disease [40]. The difference in assortativity by contact type and time-period could be used to develop and assess intervention plans based on schedules or seating arrangements, similar to the approach for assessing the effect of various vaccine coverages based on vaccination assortativity within a high school network [41].

Our findings further support that the method of collecting contact data affects the estimated network [33, 34] and could have significant impact on network analysis and in turn infectious disease modeling [42]. The necessary level of detail for modeling is unclear; preliminary work indicates that aggregated contact network data may provide enough detail for accurate transmission model predictions [35] but does not indicate the level of completeness needed for accurate predictions. Use of sensors to collect contact data is especially useful if either short duration contacts are of interest or longer duration contacts are of interest but the study population does not provide accurate self-report data. A combination of data sources provides detail and the ability to combine information. The contact frequency and mixing from Log data (Fig 5) and estimated proximal talk/touch (Fig 7) are similar but estimating the difference (possibly due to greater tendency to self-report contact with friends) and also estimating the less assortative proximal contacts relies on wireless sensor data.

There are limitations to the study. We truncated the number of self-reported contacts to 56. There were 16 who reported exactly 56 contacts. The mean degree for the 19 students who listed more than 56 contacts using the margins was 73. If these 16 failed to report extra contacts, assuming a mean degree of 73, this would be 2% of the total reported contacts which would have minimal effect on the estimates or findings. During the one-day study, we identified 10 malfunctioning WRENs. Also, physical interference between WRENs could have occurred in some instances—resulting in under-reporting of contacts. These errors were minimal and likely did not impact the results presented in the paper.

## Conclusion

Our findings reflect contact rates and mixing patterns for one middle school– 7^th^ and 8^th^ grade in Utah that contribute to an every-increasing body of knowledge and data collections on social contact. Use of WRENs and self-report logs provide complementary insight on in-school mixing patterns and estimates of contact frequency and allow estimation for types of contact. Subsequent work will be undertaken to apply these methods and assess the variability of findings across different week days, seasons, and schools. Contact data from other schools—including elementary and high schools will contribute to our understanding of mixing in the school setting. Future work must also use models of infectious disease transmission to study the impact of variation in contact and mixing among school settings on transmission.

### Ethics

Methodology for this study, including participation consent procedures for minors and data collection and storage protocol, was approved both by the University of Utah Institutional Review Board (IRB_00051285) and under the US Centers for Disease Control and Prevention IRB authorization agreement. The school district and principal approved the study prior to deployment, and teachers, students and parents were given the opportunity to opt out of the study at any time. De-identified datasets were used in the analyses presented here.

## Supporting Information

S1 Dataset Student information. (CSV)

S2 Dataset WREN data. (CSV)

S3 Dataset Log data. (CSV)

S1 Text Data description. (DOCX)
