# Supplementary material for: Estimates of Social Contact in a Middle School Based on Self-Report and Wireless Sensor Data
Source: PLoS One. 2016 Apr 21;11(4):e0153690. doi: 10.1371/journal.pone.0153690 (PMC4839567; doi:10.1371/journal.pone.0153690)
Supplement: S1 Text — (DOCX) [file pone.0153690.s004.docx]

Data Description

For details of the WREN data cleaning see supplementary information in Toth, D.J., et al., *The role of heterogeneity in contact timing and duration in network models of influenza spread in schools.* J R Soc Interface, 2015. **12**(108): p. 20150279.

Unique initials from a list combining those from the student roster and from the reported contacts in the Log were numbered. These numbers are included in the supplementary data instead of actual initials.

Information about the students and their ID number represented in the contact data is provided in S1 Dataset (Student information). The column labeled “id” corresponds to the id’s found in both the WREN and Log contact data sets; the entry is NA if the data were unusable or the student did not participate. The column labeled “grade” is the school grade of the student (7 = 7^th^ grade, 8 = 8^th^, and grade, 99 = unknown). The column labeled “gender” provides the gender of the student if known (0 = male, 1 = female, and 99 = unknown). The student’s initials number (“initialsNum”) are provided and whether or not they had a unique signature (unique = 0 or 1). The student’s post-processed assigned lunch (1 or 2, 99 if unknown) is also provided.

The WREN contact data on which the main results of our manuscript were generated consist of four columns and one row for each uninterrupted pairwise contact during the school day in S2 Dataset (WREN data). The first two columns (labeled id1 and id2) identify the two students; the third column (labeled startTime) is the time of day the contact began, in units of 19.53125 seconds (since midnight); the fourth column (labeled duration) is the duration of the uninterrupted contact, in units of 19.53125 seconds. We processed these to aggregate contacts by time period based on the school schedule (below).

The contact Log data on which the main results of our manuscript were generated consist of six columns in S3 Dataset (Log data). The “id” and “contactId” match the Student Information and WREN data. The “id” is the person who completed the Log. The “contactGender” and “contactGrade”, are what was reported. The “contactInitialsNum” is provided. If the gender, grade, and initials are unique and that person wore a WREN, their “contactId” is provided.

School Schedule

1^st^ Class 8:25 – 9:16

2^nd^ Class 9:20 – 10:11

3^rd^ Class 10:15 – 11:06

1^st^ Lunch 11:06 – 11:36

4^th^ Class (1^st^ Lunch students) 11:40 – 12:31

4^th^ Class (2^nd^ Lunch students) 11:10 – 12:01

2^nd^ Lunch 12:01 – 12:31

5^th^ Class 12:35 – 1:36

6^th^ Class 1:30 – 2:21

7^th^ Class 2:25 – 3:15
